# Nonleaching Biocidal N-Halamine-Functionalized Polyamine-, Guanidine-, and Hydantoin-Based Coatings

**DOI:** 10.1021/acs.iecr.4c00320

**Published:** 2024-03-27

**Authors:** Lev Bromberg, Beatriz Magariños, Angel Concheiro, T. Alan Hatton, Carmen Alvarez-Lorenzo

**Affiliations:** † Department of Chemical Engineering, Massachusetts Institute of Technology, Cambridge, Massachusetts 02139, United States; ‡ Department of Microbiology and Parasitology, Facultad de Biología, CIBUS, Universidade de Santiago de Compostela, 15782 Santiago de Compostela, Spain; § Department of Pharmacology, Pharmacy and Pharmaceutical Technology, I+D Farma Group (GI-1645), Facultad de Farmacia, Instituto de Materiales (iMATUS), and Health Research Institute of Santiago de Compostela (IDIS), Universidade de Santiago de Compostela, 15782 Santiago de Compostela, Spain

## Abstract

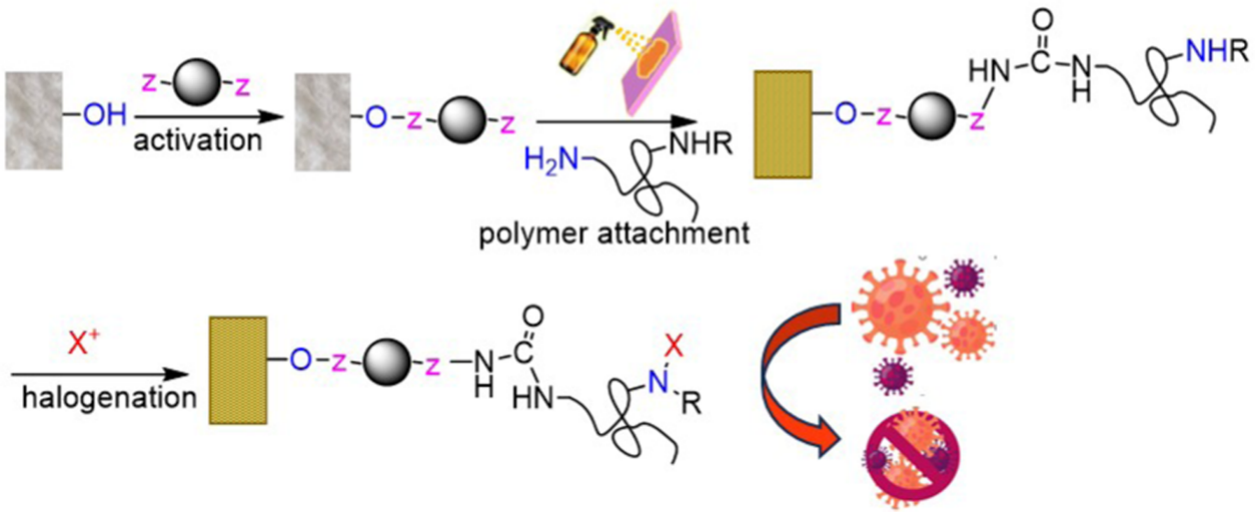

Fibrous materials with inherent antimicrobial properties can help in real-time deactivation of microorganisms, enabling multiple uses while reducing secondary infections. Coatings with antiviral polymers enhance the surface functionality for existing and potential future pandemics. Herein, we demonstrated a straightforward route toward biocidal surface creation using polymers with nucleophilic biguanide, guanidine, and hydantoin groups that are covalently attached onto a solid support. Biocidal poly(*N*-vinylguanidine) (PVG) and poly(allylamine-*co*-4-aminopyridine-*co*-5-(4-hydroxybenzylidene)hydantoin) (PAH) were introduced for coating applications along with commercially available polyvinylamine (PVAm) and poly(hexamethylene biguanide) (PHMB). Nonleaching coatings were created by first fabricating bifunctional siloxane or isocyanate precursor coatings on the cotton, nylon–cotton, and glass fiber fabric, followed by the polymer attachment. The developed grafting methods ensured the stability of the coating and the reuse of the material while maintaining the biocidal properties. Halogenation of polymer-coated fabric was conducted by aqueous solutions of sodium hypochlorite or in situ generation of hypobromous acid (HOBr), resulting in surfaces coated by N-halamines with high contents of active > N–Cl or > N–Br groups. The polymer-coated fabrics were stable in multiple laundry cycles and maintained hydrophilic character after coating and halogenation. Halogenated polymer-coated fabrics completely inactivated human respiratory coronavirus based on a contact-killing mechanism and were shown to be reusable after recharging with bromine or chlorine.

## 1 Introduction

Demand for antimicrobial agents and antimicrobial textile finishing has grown dramatically since the advent of the COVID-19 pandemic. An ideal biocidal textile material should be capable of inactivating a broad range of microorganisms, including human respiratory coronavirus, and durable to repeated washings and can be readily recharged in laundering or disinfection processes. Cationic polymers, such as polyvinylamine (PVAm) and polyethylenimine (PEI), are often applied in coatings of fibrous materials, sorbents, and membranes for improvement of antifouling, biocidal, oil–water separation, and dye and heavy metal ion removal capabilities.^1−6^ The polycationic nature of polyamines determines the ease of their deposition onto charged surfaces by the layer-by-layer (LbL) technique.^7,8^ However, polyelectrolyte layers are prone to dissociation in aqueous environments while the creation of nonleaching, covalently bonded coatings is necessary for higher durability, long-term stability, and regenerability.^9^ Covalent grafting of hydrophilic polymers on chemically inert surfaces may require plasma- or radiation-induced or photochemical surface treatment methods. Such methods typically exploit surface oxidation to render the surfaces hydrophilic and introduce reactive groups to which the coatings can be linked. These methods are tedious and require specialized equipment and several steps. Alternatively, polymer grafting can be accomplished via functionalization of the surface of the inert material by formation of reactive moieties, such as hydroxyl or carboxylic groups, C–H insertion cross-linking (CHic), and others, which can generate a wide range of polymer layers and microstructures on a broad spectrum of surfaces.^3,10,11^

Covalent grafting of polycations with primary and secondary amino groups onto engineered surfaces endowed with groups containing active hydrogen is readily accomplished by using the amine moieties with or without surface activation. Previously, we have demonstrated that spray-coating of the nylon–cotton (NYCO), rayon, and poly(p-phenylene terephthalamide) (Kevlar 119) fibers pretreated with phosphoric acid with isocyanate resulted in covalent bonding of the resulting polyurethane with the hydroxyl groups on the fiber surface.^12^ The resulting nonleaching coating accelerated the degradation of chemical threats, leading to the development of self-decontaminating textiles, gloves, and filters. In the present work, we applied methods of reactive finishing toward designing nonleaching polyamine coatings with biocidal activity. Specialty textiles and air and water filters with fibrous surfaces, wherein the coronavirus cannot remain infectious, could make a difference in epidemiology by reducing surface contamination after microbial deposition.^13−17^

**Figure 1:**
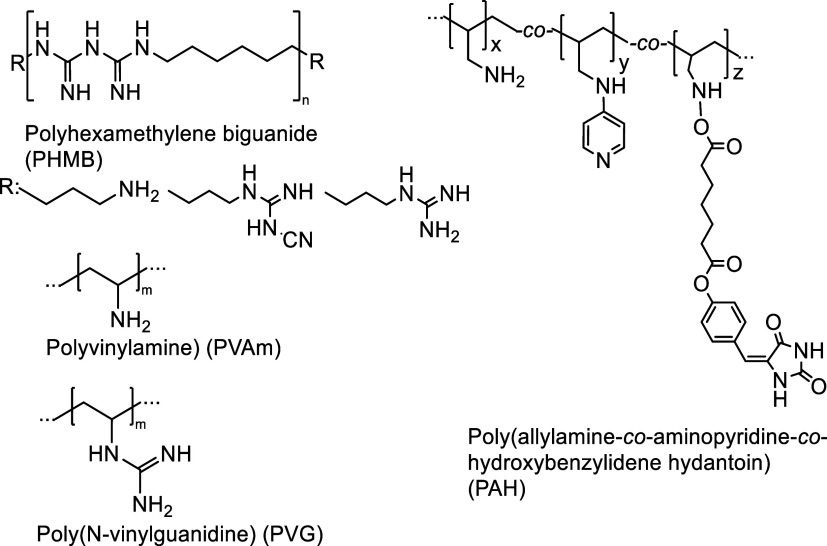
Polyamines utilized in bactericidal coatings in this work.

Polyhexamethylene biguanide (PHMB), polyvinylamine (PVAm) and its derivative poly(*N*-vinylguanidine)(PVG), and hydantoin-modified poly(4-vinylpyridine-*co*-allylamine) (PAH) (Figure 1) chosen for the covalent attachment herein are all polycationic and contain multiple >N–H groups in their structures, which are convenient handles for the activation and/or the polymer attachment. Furthermore, the contents of the >N–H groups per chain of PVAm, PVG, and PAH are among the highest among all other reported polymers, and hence, the payload of the oxidizing N-halamine > N-X (X = Br, Cl) groups after halogenation of these polymers is also among the highest.^18^ Coating combining a positively charged polymer and N-halamine can kill microorganisms on contact.^3,19^

Even though the effectiveness of polymeric N-halamine-derived coatings has been extensively demonstrated,^9,20−31^ their deposition onto engineered surfaces typically involves simple adsorption or “pad–dry” techniques or methods involving intricate polymer design or in situ polymerization. The resulting coatings either lack covalent bonding between the material surface and the N-halamine moiety or are very complex to fabricate on an industrial scale. Alternatively, N-halamine precursors can be bonded to substrates via different tethering groups, such as epoxide, diol, or siloxane, but such precursors require multistep synthesis targeting specific functionalities, which can be used to both graft the precursor and halogenate the available >N–H groups. In the present work, we focused on covalent grafting of ready-made polycations rich in amine groups, including commercially available ones (PHMB, PVAm) by methods resembling those of functional fabric finishing.

PHMB, PVG, and PAH are cationic and biocidal without halogenation, but their biocidal activity is dramatically enhanced by chlorination or bromination, yet the halogenated species possess an acceptable safety profile vs mammalian cells.^18^ PHMB has been reported to kill up to 99% of coronavirus when coated onto cotton fabrics.^32−34^ Halogenation by either chlorine or bromine can be effective, although the brominated surface would be expected to be more bactericidal than an analogous chlorinated one.^28,35^

## 2 Experimental Section

### 2.1 Materials

Cotton fabric (cotton 400, abbreviated in this work as C1) and standard reference WOB (without optical brightener) detergent were purchased from Testfabrics, Inc. (West Pittston, PA). Fabric C1 (10 cm × 10 cm coupons) was washed with boiling deionized water and dried in air and under vacuum until a constant weight was achieved prior to use. Coupons (22 × 30 cm) of lint-free 100% cotton cloth, woven with tight weave (weight, 190 g/m^2^, abbreviated in this work as C2), and standard Army Combat Uniform fabric woven using blended yarns of 50% nylon staple–50% cotton fiber (weave ripstop, untreated, weight 220 g/m^2^, abbreviated NYCO) were obtained from the U.S. Army Natick Soldier Systems Center (Natick, MA). The C2 and NYCO textile fabrics were carefully cleaned by washing in a 2% nonionic detergent (Alfonic 810-6, linear alcohol ethoxylate, Sasol North America, Lake Charles, LA) at about pH 7 and 40 °C for 30 min, then rinsed several times with deionized water, and air-dried. Wetlaid filament fiberglass nonwoven fabric (Craneglas series 230 (weight, ∼20 g/m^2^) and 333 (∼200 g/m^2^), abbreviated in this work as G) was received from Mativ (Pittsfield, MA) and used as received. Craneglas fabric consists of fiberglass filaments and contains about 7% polymer binder; the surface contains a multitude of hydroxylic groups. Triphenylmethane-4,4′,4″-triisocyanate (TPMTI) was received as a 27% solution in ethyl acetate (Desmodur RE, NCO group content, 9.3 ± 0.2%) from Covestro AG (Germany). Polyvinylamine (PVAm) was obtained from the commercial product Lupamin 9095 (BASF, Germany) as an aqueous 20–22 wt % solution with a nominal molecular weight of 340 kDa and pH = 7–9. Prior to use, PVAm was fully hydrolyzed using 3 M HCl and purified by dialysis (membrane MWCO, 12–14 kDa) in deionized water and lyophilization. PVAm was then reacted with cyanamide to result in poly(*N*-vinylguanidine) (PVG). The synthesis, characterization, and properties of PVG (average *M*_w_ 340 kDa by GPC) have been described in detail previously.^36^ (C_3_H_7_N_3_)_*x*_, found (calc): C 42.15 (42.3); H, 9.6 (8.29); N, 49.1 (49.37). Poly(hexamethylene biguanide) (PHMB) was obtained from Lonza Consumer Care (South Plainfield, NJ), supplied as a 20 wt % aqueous solution (Cosmocil CQ) with a reported *M*_w_ of 2700 and a polydispersity of 1.9. The solution was dialyzed against deionized water (MWCO = 2 kDa) and lyophilized to dryness. After dialysis, the *M*_w_ and polydispersity were 2810 and 1.69, respectively. That is, the PHMB chain on average consisted of 7.3 repeating units. (C_8_H_19_N_5_)_*x*_, found (calc.): C, 51.9 (51.86); H, 11.2 (10.34); N, 37.6 (37.80).

Poly(allylamine-*co*-4-aminopyridine) (PAAm-APy) was synthesized from poly(allylamine) hydrochloride (PAAm.HCl, average *M*_w_ 60 kDa by GPC, Sigma-Aldrich Chemical Co., St. Louis, MO) converted to PAAm.^18^ PAAm was modified with 1-(3-amino-3-oxopropyl)-4-cyanopyridin-1-ium chloride to result in poly(allylamine-*co*-4-aminopyridine) (PAAm-APy). PAAm-APy chains were further modified with 5-(4-hydroxybenzylidene)hydantoin (HBH) moieties to result in a terpolymer (PAH), with the PAAm/APy/HBH monomer molar ratio estimated to be 9:50:41 using ^1^H NMR. ^1^H NMR (400 MHz, DMSO-*d*_6_): δ 1.25–1.45 (m, CH), 2.69 (m, CH_2_ α to −N), 2.88, 3.15 (m, CH_2_ α to −N-CR), 6.0, 6.1 (m, secondary −NH), 6.5 (m, pyridine), 7.0 (m, CH pyridine), 7.26 (m, benzene), 7.9, 8.5 (m, 2H pyridine), 9.3 (m, amine). The PAH polymer (*M*_w_ = 60 kDa by GPC) was soluble in water, methanol, and N,N-dimethylformamide. PHMB, PVG, and PAH samples were kept as dry powders in desiccators prior to use. Ultrapure water (18 MΩ cm^–1^, Milli-Q) was used throughout. All other chemicals were of the highest purity available and were obtained from Sigma-Aldrich Chemical Co.

### 2.2 Equipment

FTIR measurements were conducted by using a Nicolet 8700 FTIR spectrometer (Thermo Scientific) and a Bruker α II FTIR spectrometer with a diamond crystal attenuated total reflectance accessory (ATR). FTIR spectra were measured in KBr tablets at 2 cm^–1^ resolution with 64 scans, or as well as subjected to ATR, wherein a total of 128 spectra (2 cm^–1^ resolution) were acquired and averaged for every sample. ^1^H NMR spectra of polymer solutions in D_2_O or DMSO-*d*_6_ were taken by using a Bruker Avance-III HD Nanobay spectrometer operating at 400.09 MHz. Electronic absorption spectra were measured using a Cary 60 UV–vis spectrophotometer (Agilent). XPS spectra were acquired using a PHI Versaprobe II XPS instrument with a scanning X-ray source and a UV lamp (Physical Electronics, Inc.). Peak assignments were performed using built-in instrument software. Elemental analysis of solids was performed in an EPA-certified laboratory.

### 2.3 Fabric Modification by Polymers

The first route of fabric modification by polymers (*process A*) involved treatment of the fabrics with (3-glycidyloxypropyl)trimethoxysilane (GPTMS), followed by polymer attachment (*process B*). GPTMS (0.3 M solution in 30/70 v/v ethanol/water mixture) was prehydrolyzed by adding 0.1 M HCl dropwise up to a 4% v/v concentration to obtain a final pH 4. The reaction was carried out for 4 h under vigorous stirring at room temperature shortly prior to the application. The resulting sols were impregnated into the textile fabric coupons and passed through a two-roll laboratory padder TD122 (ATI Corporation) to achieve about 70% of wet pick-up. After drying at 80 °C for 10 min, the padded samples were sprayed with a 1 or 2 wt % (PVAm, PVG) or 2 to 4 wt % (PHMB, PAH) aqueous solution of a given polymer as described above. The padded and coated coupons were then cured at 130 °C in a gravity convection oven for 4 min, air-dried at 40 °C for 48 h, and kept in desiccators prior to further use. For the polymer attachment by process *B*, the following two-step procedure was applied. Dry, weighed fabric coupons were placed in a Petri dish. Solution of triphenylmethane-4,4′,4″-triisocyanate (TPMTI) in ethyl acetate was loaded into a Dynalon Quick Mist 16 oz spray bottle and repeatedly sprayed for 5–10 s on one side of the fabric. The sprayed solution droplets rapidly soaked into the fabric. The sprayed coupons were dried under a stream of nitrogen at room temperature for 2–4 h until a constant weight was achieved, and the weight gain was calculated to estimate the effective NCO group loading using the manufacturer’s specifications for the triisocyanate solution. In the control experiments, the TPMTI-coated coupons were cured at 60 °C for 4 days. The free NCO content was determined by butylamine back-titration according to EN ISO 14896. The second step of the fabric treatment was conducted by spraying with a 1 or 2 wt % (PVAm, PVG) or 2 to 4 wt % (PHMB, PAH) aqueous solution of a given polymer. The polymer deposition was repeated several times as needed to achieve the targeted content of a given polymer with the wet coupons weighed after spraying. The treated fabric coupons were then kept at 60 °C for 2–3 days for curing and drying, at which point the coupons reached constant weight. The resulting dry coupons had weight gain due to the polymer attachment varied from 1 to 5 wt %. In the control experiments, fabric sheets that had not undergone treatment with the isocyanate were subjected to polymer deposition as described above.

#### 2.3.1 Chlorination Procedures

Polymer-modified fabric coupons were soaked in a NaOCl solution (Sigma-Aldrich, available chlorine, 4–5%, pH adjusted to 7) for 1 h at room temperature. The chlorinated fabric samples were washed thoroughly with distilled water and dried at 45 °C for 1–2 h to remove the free oxidative chlorine absorbed on the surface. The chlorinated fabric samples were subjected to quantitative elemental analysis to determine the chlorine content by microwave digestion ICP-MS.

#### 2.3.2 Bromination Procedures

Active bromination of polymer-coated fabric was conducted through the generation of excess hypobromous acid (HOBr) in situ from bromine and sodium hydroxide. A stirred, temperature-controlled reactor charged with a 3.2 M aqueous solution of NaOH (1 L) was equilibrated at 10–15 °C using an ice bath. Bromine (56 mL, 5 mol) was added dropwise while stirring, and the contents rapidly became dark orange to brown in color. The pH of this solution was adjusted to 6.8 with 4 N acetic acid, and the polymer-modified fabric coupons were suspended while stirring. The coupons were immersed in the brominating solution for 30 min at 25 °C. The resulting brominated fabric was carefully separated from the solution and washed on a glass filter by acetone and deionized water until the runoff water produced no color response using a Taylor Complete DPD Test Kit (Taylor Water Technologies LLC, Sparks, MD). The fabric was then again rinsed and dried over anhydrous sodium sulfate. Each coated fabric species was characterized by an elemental analysis for the total bromine content.

### 2.4 Fabric Testing

#### 2.4.1 Polymer Attachment Stability Test

Fabric samples with polymers deposited were stamped into circular coupons (1.5 cm diameter), weighed, and placed into 0.15% (w/v) WOB detergent solution (50 mL). The treated fabric samples were then laundered 15 cycles, according to the AATCC Test Method 135-2000. After the test, the fabric coupons were removed from the test solution, rinsed with deionized water, and dried in air until a constant weight at 45 °C was achieved. The polymer deposition stability, *DS*, was calculated as follows
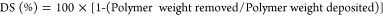


#### 2.4.2 Polymer Attachment Durability Test

The AATCC Test Method 61-1996 was applied to evaluate the durability of the C2 and NYCO fabric containing 3 wt % PHMB attached via *process A* or *B* against cycling laundering. Fabric coupons of 1.5 × 1.5 cm^2^ size were put into 150 mL of 0.15% (w/v) WOB detergent solution at 49 °C for 15 laundering cycles. Then, the samples were washed with tap water and air-dried at room temperature for antimicrobial action testing against *Staphylococcus aureus* and *Escherichia coli*.

#### 2.4.3 Halogen Release Tests

Chloride-free phosphate buffer (PB, 0.01M, pH 7.4) was prepared from 10 mM KH_2_PO_4_, 10 mM K_2_HPO_4_, 0.27 mM KNO_3_, and 13.7 mM NaNO_3_ solutions. Weighed amounts of circular polymer-modified fabric coupons (1.5 cm diameter) with known halogen contents were suspended in 50 mL of PB and placed in light-safe sealed tubes. The mixture was shaken at room temperature for a specified time, 1.5 mL aliquots were withdrawn intermittently, and the supernatant was assayed for (i) oxidative halogen content using a Hanna PCA330 ORP analyzer (Hanna Instruments, Smithfield, RI) and (ii) total halogen contents using ICP-MS (NexION 2000, PerkinElmer, Shelton, CT).

Release of halogen was calculated as Rel (%) = 100 × *C*_*t*_/*C*_o_, where *C*_*t*_ and *C*_o_ are the halogen concentrations in the supernatant at time *t* and initial concentrations, respectively. The measurements were conducted in triplicate. The *C*_o_ values were calculated from elemental analysis and the mass of the dry coupon. Coupons were removed from PB after 28 days period, rinsed in DI water, and dried at 45 °C for 1 h. The halogen content remaining in each type of coupon was measured. The coupons were rinsed thoroughly and then subjected to a rehalogenation process by the procedures described above. The halogen content after rehalogenation (coating “re-charging”) was again determined for comparison.

#### 2.4.4 Sessile Drop Testing

Surface water contact angles and drop volumes were measured at room temperature in air with a relative humidity of approximately 30% using a sessile drop method with a Kruss DSA10 mk2 drop shape analyzer (Krüss, Hamburg, Germany). Pure water surface tension was determined from pendant drops having volumes of 5–10 μL and were found to be 73 mN/m. Advancing contact angles (CAs) and drop volumes were measured immediately after the drop was placed on the surface with a small syringe and needle. The average initial drop volume was 1.2 μL. The contact angle and drop volume were taken through the water phase at 1 s interval. The drop shape analysis software reported an average value of the measured data. Contact angles (CAs) were obtained at least at four separate locations of each coupon sample. Significant hysteresis was observed from the difference between advancing and receding water CAs due to droplet wicking and surface reactivity. Four measurements from different coupons were taken for each surface treatment.

#### 2.4.5 Mechanical Testing

The tensile strength and the elongation at break of polymer-modified C2 and NYCO fabric samples were evaluated on a Shimadzu AGS-X Universal Tester (Shimadzu Europa GmbH, Duisburg, Germany), according to ASTM D5034-21. The equipment was fitted with a 1 kN cell load. The speed of the sample elongation was 300 mm/min and the gap was 75 mm.

### 2.5 Testing of Biocidal Properties

#### 2.5.1 Human Coronavirus Inactivation

Human coronavirus 229E (ATCC VR-740) was grown and propagated in the human embryonic L-132 cell line (human lung epithelium; ATCC: CCL5). The maintenance medium consisted of minimum essential medium (MEM, Sigma-Aldrich, St. Louis, MO) without fetal bovine serum containing 100 IU/mL penicillin and 100 μg/mL streptomycin. The neutralizing broth was soya casein digest lecithin polysorbate neutralizing broth (SCDLP, Sigma-Aldrich, St. Louis, MO). Viruses were purified by centrifugation to remove cell debris, followed by PEG precipitation. Virus stock was stored at −80 °C. Infectious virus titers were determined as log_10_ 50% tissue culture infective doses (TCID_50_) in confluent cells in 96-well microtiter plates. *Testing of fabric coupons* (ISO 18184:2019). Weighed circular fabric coupons (diameter 5 cm) modified with 5 wt % polymers were sanitized by 70% ethanol, air-dried, and inoculated with 200 mL of diluted 229E virus. Control sterilized fabric coupons without the polymer added were treated identically in separate sterile plates. The coupons were inoculated at 95% relative humidity and 23 °C. Immediately after the inoculation of the virus, the SCDLP broth was added to the control samples. At 0.5 h, the SCDLP broth was added to the polymer-coated and control samples to recover the remaining virus. The wash-out solutions were serially diluted, and the infectious titer (antiviral activity value, AAV) and inactivation rate (IR, %) of the recovered virus were determined by the TCID_50_ assay
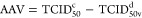

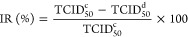
where TCID_50_^*c*^ is the average TCID_50_ immediately after inoculation of the control coupons and TCID_50_^d^ is the average TCID_50_ after 0.5 h contact time with the polymer-coated coupons. In a separate series of experiments designed to demonstrate the coating’s recharge with halogen and reuse, the polymer-coated coupons were recovered from the SCDLP broth, rinsed with deionized water, and steam-sterilized at 121 °C for 30 min. The coupons were then halogenated as described above and subjected to coronavirus killing tests.

#### 2.5.2 Quantitation of Bactericidal Properties after Laundering

The effects of laundering and halogenation on the bactericidal properties of the polymer-modified C2 and NYCO fabrics were evaluated by a quantitative test method (dynamic shake), according to ASTM E 2149-01 and AATCC Test Method 100–1999 with Gram-positive *Staphylococcus aureus* (ATCC 6538) and Gram-negative bacteria *Escherichia coli* (ATCC 11229), utilized as test organisms. Bacterial strains were cultured in Mueller-Hinton Broth (MHB, 37 °C, 18 h). Then, the cultured bacteria in MHB were diluted with turbidimetry measurements at 625 nm to a 0.5 McFarland (approximately 1 × 10^8^ cfu/mL) and then to a final inoculum concentration. The polymer-coated and control fabric coupons were sterilized in an autoclave at 121 °C for 15 min. The weighed coupons and 5 mL of the diluted bacteria suspension were added to a flask containing 70 mL of phosphate-buffered saline, which was then shaken at 150 rpm for 18 h at room temperature. Next, bacterial suspension collected from each flask (1 mL) was diluted 10, 100, and 1000 times and the diluted samples were inoculated onto agar medium for 24 h at 37 °C, and colonies were counted. Each measurement was conducted in triplicate. The bactericidal properties were evaluated by the reduction percentage of colonies between the treated and control samples. The antimicrobial activity was expressed as Reduction (%) = 100 × *P*/*Q*, where *P* and *Q* are the numbers of surviving colonies in the flasks containing the polymer-coated and control samples, respectively.

## 3 Results and Discussion

### 3.1 Characterization of Coated Fabrics

**Figure 2:**
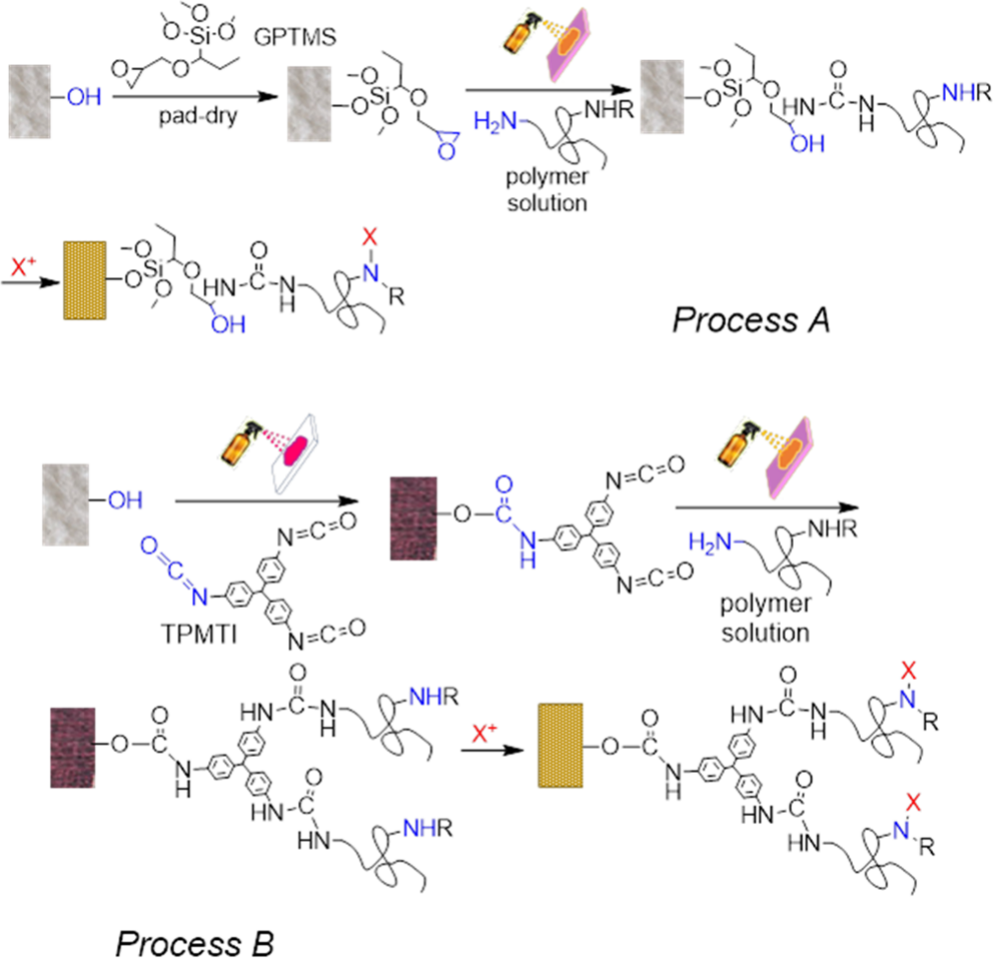
Schematic of the coating route. *In process A*, the fabric is treated with functional sol of (3-glycidoxypropyl)trimethoxysilane (GPTMS) by a pad–dry technique, followed by spraying with an aqueous polymer solution with subsequent drying and curing. The coated fabric is halogenated by hypobromous acid or hypochlorite that releases an oxidizing X^+^ agent (X = Br or Cl). *In process B*, the fabric is sprayed with a solution of triphenylmethane-4,4′,4″-triisocyanate (TPMTI) in ethyl acetate. Followed by gentle drying, the fabric is sprayed with an aqueous polymer solution with subsequent drying and curing.

The coating routes are schematically depicted in Figure 2. *Process A* involved impregnation of the fabric with functional sol of (3-glycidoxypropyl)trimethoxysilane (GPTMS) by a pad–dry technique. Each silanol (Si–OH) group obtained in the GPTMS hydrolysis catalyzed by HCl^37^ can react with other Si–OH groups to form stable siloxane bonds (Si–O–Si) or with the hydroxyl groups belonging to the cellulose fibers on the cotton or NYCO fabric surface (C) to form a stable C–O–Si bond.^37−39^ The siloxane sol of GPTMS possesses epoxide groups that undergo a ring-opening reaction with the incoming primary amine functionalities. *Process B* for the covalent polymer attachment included spraying the substrate with the multifunctional isocyanate (TPMTI) solution, which provided an amide link to the underlying glass/binder, cotton, or NYCO surface while still exposing excess active isocyanate groups toward further modification by the incoming polymer with amine groups capable of forming a urea link with the TPMTI on the fabric surface. The final step of *process B* (Figure 2) included fabric spraying with an aqueous polymer solution with subsequent drying and curing. Unreacted amine, imine, guanidine, or hydantoin groups on the polymer-coated surface were then either chlorinated by sodium hypochlorite or brominated by the in-situ-formed hypobromous acid/hypobromite anions containing reactive halogen atoms in its +1 oxidation state (X^+^) (Figure 2).

**Figure 3:**
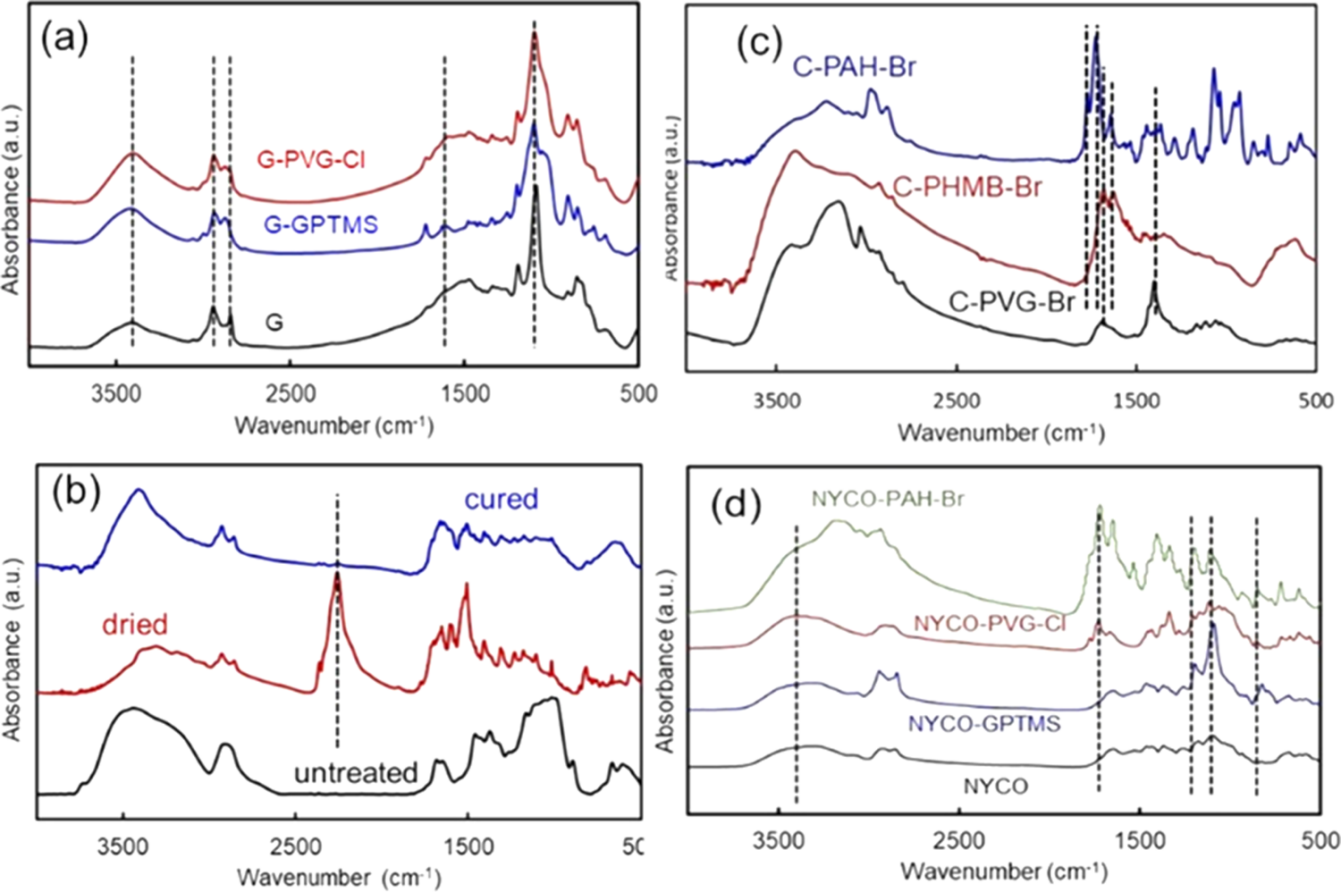
FTIR spectra illustrating the chemistry of the coatings. (a) The untreated glass fiber (G) fabric, the same fabric coated by GPTMS by the pad–dry method (*process A* in Figure 2), and the same G-GPTMS fabric grafted by PVG followed by chlorination and drying. Vertical lines at 3350, 2940, 2840, 1635, and 1085 cm^–1^ indicate the O–H stretching vibration of the hydroxyl groups, asymmetric and symmetric stretching of methyl and −CH_2_– groups, N–H bending vibration, and asymmetric stretching of Si–O–Si groups, respectively. (b) FTIR spectra of untreated cotton (C1) fabric, the same fabric coated by TPMTI by spraying and drying of the coupon in nitrogen flow at room temperature (dried), and the same fabric coated by TPMTI followed by curing at 60 °C for 4 days (cured) are shown, illustrating *process B* in Figure 2. Vertical dotted line at 2275 cm^–1^ indicates the free NCO group stretching vibration peak (nNCO). (c) FTIR-ATR spectra of the cotton C1 fabric modified by TPMTI and by brominated polymers PVG-Br, PHMB-Br, and PAH-Br are shown. Vertical lines at 1771, 1724, 1680, 1625, and 1410 cm^–1^ designate vibrations of the following groups: brominated amide group of hydantoin, brominated imide group of hydantoin, C=N stretch, N–H bending, and stretching of the aromatic double bonds in TPMTI, respectively. (d) FTIR-ATR spectra of the NYCO fabric modified with GPTMS (uncured) and the same fabric modified by GPTMS and brominated or chlorinated polymers (PAH-Br, PVG-Cl) are shown. Vertical lines at 3300, 1190, 1085, and 8127 cm^–1^ designate −OH stretch, CO stretch, CH_2_ wag, SiO stretch, CH_3_ rock, CH_2_ rock, and SiO stretch, respectively.^39^

**Figure 4:**
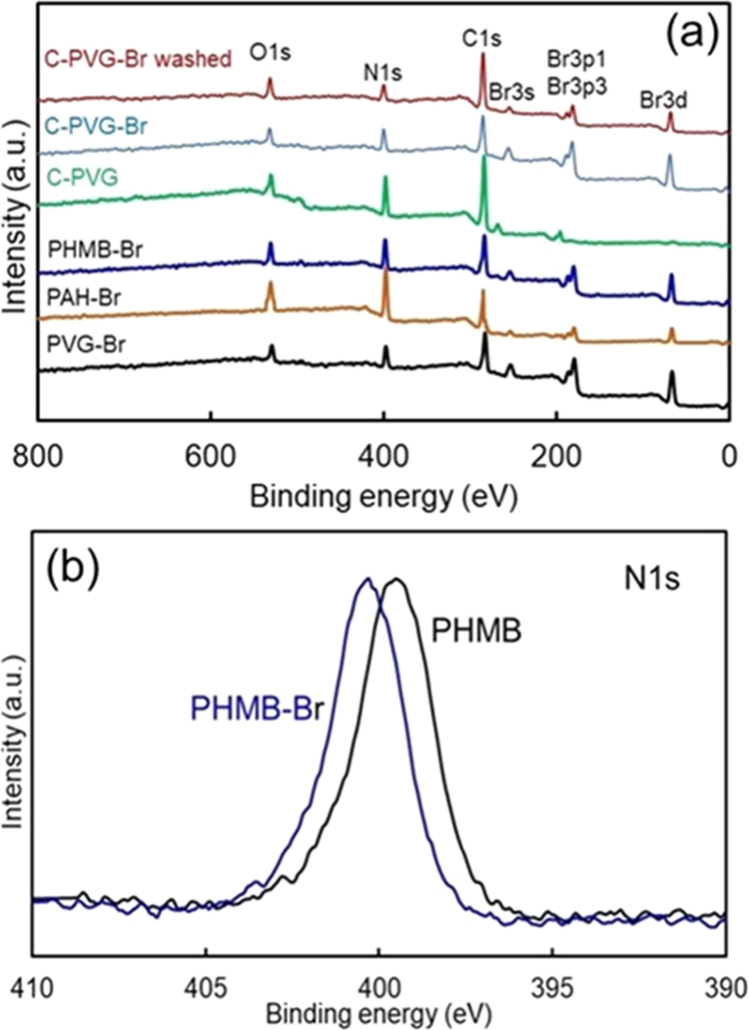
(a) XPS survey scans of the brominated polymers (PVG-Br, PAH-Br, PHMB-Br) and cotton (C) fabric that underwent covalent attachment of PVG (C-PVG) and then was brominated (C-PVG-Br), and of the same brominated fabric that was subsequently washed in chlorine-free PB at pH 7.4 (C-PVG-Br washed). (b) High-resolution nitrogen (N 1s) spectra of the cotton C1 fabric coated with PHMB and PHMB-Br.

The chemical processes occurring on the fabric surfaces were illustrated by FTIR spectroscopy (Figure 3) and XPS (Figure 4). All fabrics under study were hydrophilic and featured broad peaks in the 3400–3200 cm^–1^ range, characteristic of stretching vibrations of the −OH groups (Figure 3).

Spectra of glass fiber and NYCO fabrics treated with GPTMS and polymers followed by halogenation show characteristic bands corresponding to hydroxyl, silanol, siloxane, methoxy, propyl, and glycidoxy groups (Figure 3a,d). The spectra of cotton C1 fabric coated by a TPMTI solution followed by simple drying by nitrogen flow at room temperature resembled those of the dry TPMTI, as the TPMTI isocyanate covered the surface completely (Figure 3b). An intense peak characteristic of the antisymmetric stretching vibration of the −NCO groups at 2275 cm^–1^ was observed, indicating that simple TPMTI deposition without curing left free isocyanate groups available for reaction with the polymers to be deposited next. The peak corresponding to the free NCO groups totally disappeared in the sample cured at 60 °C due to the TPMTI wicking and reaction with the −OH groups on the cotton surface, but characteristic peaks expected for polyurethane derived from Desmodur RE appeared. These included the stretching vibration of the carbonyl of the urea group around 1656 cm^–1^, the scissoring vibration of the N–H group around 1524 cm^–1^, and the stretching vibrations of the aromatic double bonds at 1508 and 1405 cm^–1^. These observations illustrate the route for the polymer coating that was preceded by TPMTI deposition, followed by drying at room temperature only, which left free NCO groups on the surface available for the covalent attachment of the polymers (Figure 3). FTIR spectra of cotton coated by TPMTI and polymers following bromination are shown in Figure 3c. Notably, the strong scissoring vibration band of the N–H group of the unmodified PHMB around 1536 cm^–1^ ^40−44^ and the stretching vibration band of the terminal –C≡N bonds at 2170 cm^–1^ ^43,44^ disappeared after the bromination procedure due to the halogenation and hydrolysis, respectively (Figure S1). These changes reflect upon the conversion of the guanidine >N–H groups into the halamine >N–Br group.

X-ray photoelectron spectroscopy (XPS) spectra of the polymers and modified fabric enable further characterization of the halogenated materials (Figure 4). Brominated polymers were studied in more detail. Bromination procedure resulted in the appearance of distinct Br 3d peaks at 68 eV, Br 3p peaks at ∼182 and ∼189 eV and weaker Br 3s peaks at ∼254 eV. The Br 3d peak is consistent with the >N–Br bond formation. The bromine concentrations in dry PHMB-Br, PAH-Br, and PVG-Br obtained from the relative sensitivity factor (RSF)-corrected XPS were 45.6, 30.8, and 44.7%, respectively, which corresponds to the values obtained by the elemental analysis within ±15%. Likewise, chlorine concentrations obtained from the RSF-corrected XPS (spectra not shown) for chlorinated PHMB-Cl, PVAm-Cl, PAH-Cl, and PVG-Cl were 7.4, 7.8, 8.1, and 11.6%, respectively, all within 10% of the previously reported data obtained by the elemental analysis.^18^ Additional washing of the brominated cotton fabric did not change the bromine content appreciably. Halogen content determination by XPS can be affected by the coupon surface being rich in bromine, surface sensitivity of XPS, and the partial decomposition of the N–Br or N–Cl bond under ultrahigh vacuum conditions. Furthermore, the N 1s (N–H bond peak representing amine or imine groups) in the high-resolution XPS spectra of the polymers observed in the range of 398.4 to 399.2 eV (depending on the polymer) shifted by about 0.8 eV after bromination toward higher binding energy, which was consistent with the formation of N-halamine nitrogen atoms (N–Br), with Br possessing higher electronegativity than hydrogen (Figure 4b). The electronegativity values for H, Br, and N atoms are 2.1, 2.8, and 3.0, respectively, and bond energies for N–Br and N–H bonds are 243 and 391 kJ/mol, respectively.^45,46^

### 3.2 Polymer Deposition Stability

Stability of the polymer deposition on the fabrics was tested under the conditions of the standard laundering test (Figure S2). Polymer deposition via simple adsorption and the coating processes depicted in Figure 2 were compared. The adsorption technique resulted in facile dissociation and removal of 80–90% of the deposited polymer. In contrast, fabric treatment with GPTMS (*process A*, Figure 2) by the pad–dry method resembling industrial fabric finishing processes^47−49^ resulted in stable deposition of all studied polymers, with DS > 95% for all polymers attached. Likewise, the chosen multifunctional isocyanate, TPMTI, is known to be an effective cross-linker for hydroxyl-containing adhesives formulated for a range of bonding and lamination applications in a variety of industries. Our tests demonstrated that the fabric coupons with the polymers covalently attached through the reactions with TPMTI (*process B*) did not lose any significant fraction of the polymers (*DS* > 98% in all cases) after 15 laundering cycles (Figure S2). Furthermore, maintenance of the bactericidal activity by the fabric coated with a strongly bactericidal polymer, PHMB, after multiple laundering cycles is a sensitive test of the polymer attachment stability. As shown in Table S1, after incubation with PHMB-modified cotton and NYCO fabric laundered during 15 standard cycles, there was a 95.1 to 99.6% reduction in viable *S. aureus* and *E. coli*. This indicated that the fabrics coated by either process depicted in Figure 2 retained their antimicrobial activities and were durable against repeated launderings.

These tests demonstrated the efficiency of the chosen polymer coating routes and proved the concept of immobilizing water-soluble cationic polymers on insoluble supports by covalent bonding to prevent leaching. Previously reported^32−34^ pad–dry–cure treatment of the cotton and Spandex fabric with PHMB without the sol–gel reactions involved a curing process at 130 °C that apparently created a more stable bonding of PHMB to the cellulose chains than the physisorption at 60 °C and can produce a fabric capable of withstanding multiple laundering cycles without losing much of biocidal activity. However, this method may not be applicable to the combinations of other biocidal polymers and engineered surfaces.

### 3.3 Effects of Halogenation

#### 3.3.1 Halogen Release

**Figure 5:**
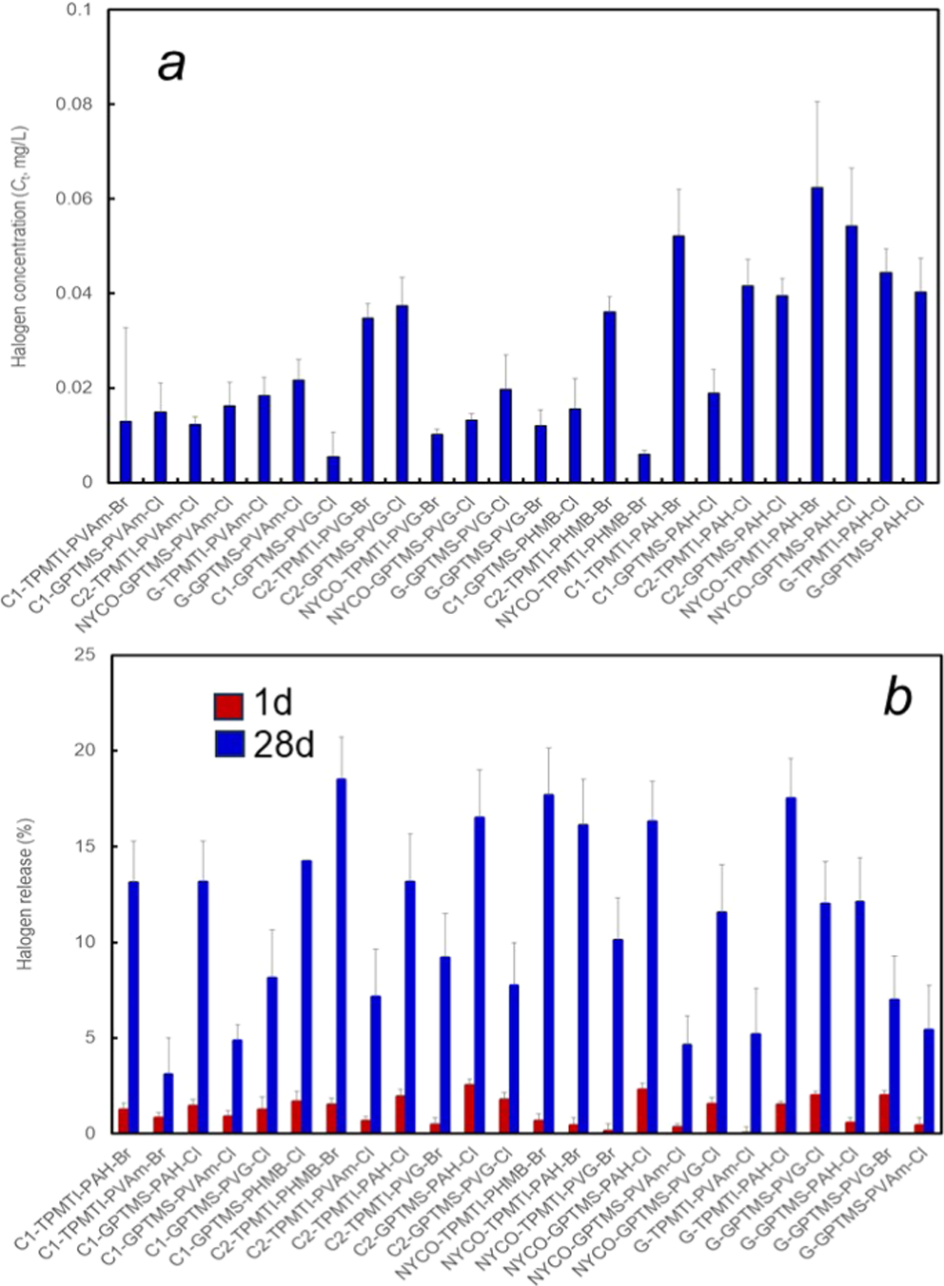
Halogen release from brominated and chlorinated cotton C1 or C2, nylon–cotton (NYCO), and glass fiber (G) fabrics coated with polymers into chloride-free phosphate buffer (0.01M, pH 7.4) at room temperature. (a) The oxidizing (positive) halogen ion concentration in the buffer solution (Ct) is shown. (b) Total halogen release relative to the initial halogen concentration in the fabric is measured after 1 and 28 days. Designations GPTMS and TPMTI indicate that the corresponding fabric was coated via either *process A* using (3-glycidoxypropyl)trimethoxysilane (GPTMS) or via *process B* using (3- triphenylmethane-4,4′,4″-triisocyanate (TPMTI). In panel (a), the samples are grouped based on the polymer attached to the fabrics, whereas in panel (b), the samples are grouped based on the type of fabric used.

Halogenation, the last step in the coating processes (Figure 2), resulted in polymer-coated fabrics that contained large loads of halogen. Total chlorine and bromine contents in the fabrics varied in 0.15–4 and 2–20 mg/g ranges, respectively, depending on the amount of the polymer deposited. At a nominal fabric weight of 200 g/m^2^, this translates into effective loads of oxidative chlorine or bromine of at least 3 × 10^20^ to 3 × 10^21^ atoms per cm^2^ of fabric. It is established that the surface concentration of oxidative chlorine as low as 5 × 10^15^ atoms/cm^2^ is sufficient to provide an antimicrobial effect.^28^ Therefore, it is evident that our polymer-coated and halogenated fabrics possessed large excess of oxidative halogen for the “contact killing”, even considering that only a fraction of that halogen would be available for contact with the microorganism at the fibrous fabric/water interface. To estimate the release of halogen from the polymer-coated, halogenated fabrics via the > N–X bond hydrolysis and dissociation, time-dependent quantitative evaluation of the oxidative and total halogen contents in the immersing solutions was conducted at the initial stage (up to 1 h), and total halogen contents were measured at 1 and 28 days since the release commencement (Figure 5). The oxidative and total halogen concentrations measured up to 1 h after the release coincided within the error of measurements (±7%). It was found (Figure 5A) that the positive halogen (Cl^+^ or Br^+^) concentrations in the immersing solutions reached up to approximately 0.08 mg/L within 1 h, which is below 0.1 mg/L level typically considered biocidal at neutral pH and is also below EPA maximum residual disinfectant level in drinking water (4 mg/L).^50^ Hence, at contact times <1 h, the release killing mechanism by the positive halogen ions dissociating from the surface of our polymer-coated fabrics can be ruled out. Of note, halogen concentrations in solutions equilibrated with fabrics modified by the PAH polymer containing cyclic hydantoin groups in its structure were generally higher than those with other polymers, which contained only amine, guanidine, and biguanide halamine groups (Figure 5a). This agrees with the well-founded observation that amine halamine is the most stable of all N-halamine bonds, dissociation constants of which decrease with the chemical structure in the order imide > amide > amine.^51−53^ Conversely, cyclical hydroxybenzylidene hydantoin moieties of PAH, each containing two imide N–H bonds capable of halogen binding, are expected to be least stable and possess higher dissociation constants when converted to the N–X bonds, which explains higher, on average, halogen concentrations released by PAH-coated fabrics in the initial period (Figure 5a). Likewise, lower dissociation or hydrolysis constants reported by the amine N-halamines probably explain the lower total halogen concentrations released after 1 and 28 days by the PVAm-coated fabrics (Figure 5b).

Significantly, up to 20% release of the total halogen after 28 days was observed due to the hydrolysis of the reactive N–X moieties of the hydrophilic polymers when the fabrics were continually immersed in water at neutral pH (Figure 5b). However, following the loss of oxidative halogen due to hydrolysis, the coupons of the coated fabrics could be recharged by exposure to dilute hypochlorite or hypobromous acid (see Experimental Section) to approximately 90–100% of the starting loadings. This indicates that the coatings were completely rechargeable.

#### 3.3.2 Mechanical Properties

**Figure 6:**
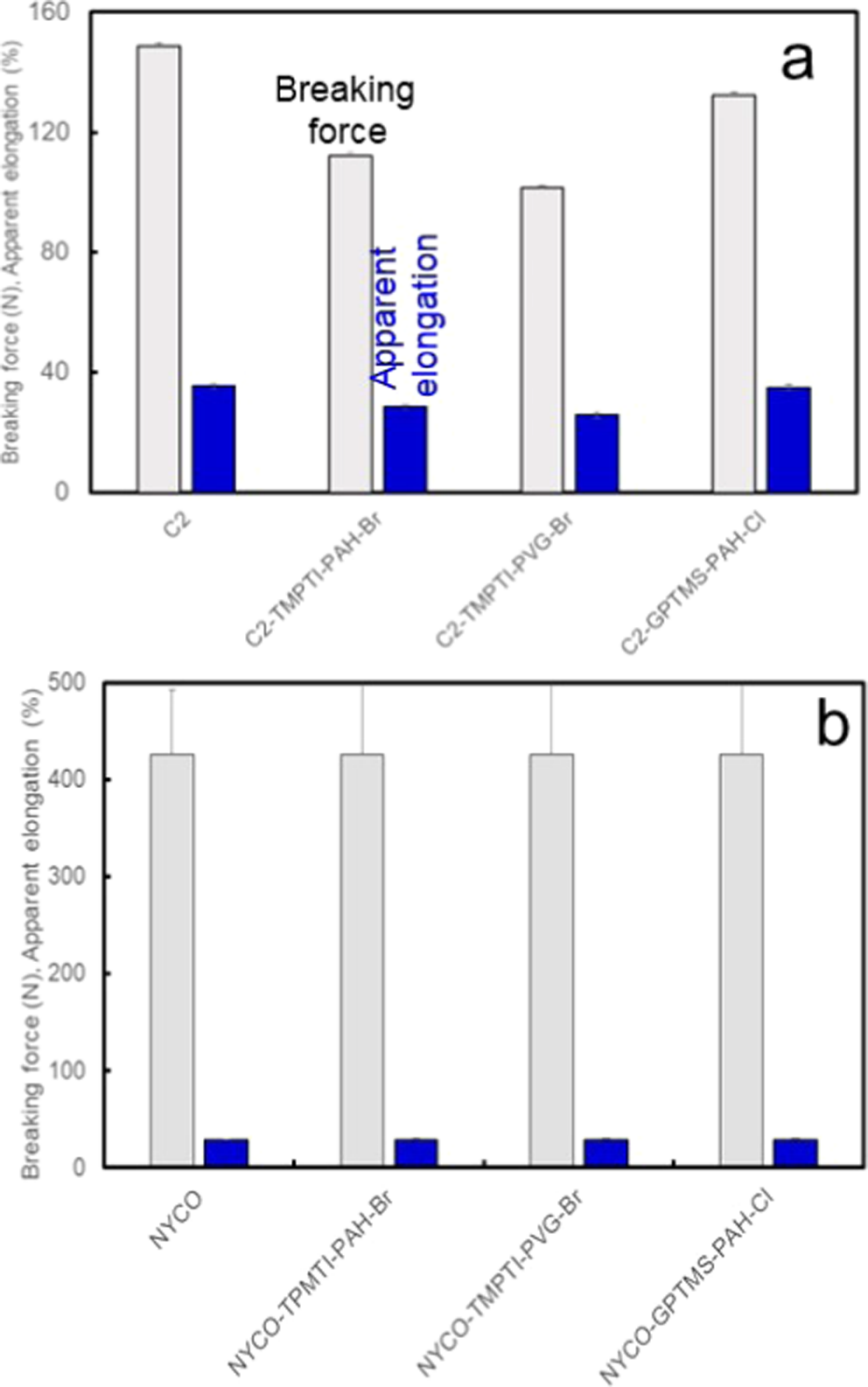
Breaking force and apparent elongation of representative cotton C2 (a) and NYCO (b) fabrics before and after coating processes and halogenation.

The effect of the coating processes on the mechanical properties of cotton and NYCO textile fabrics is shown in Figure 6. The coating and halogenation processes with TPMTI lowered the breaking force and relative elongation of the cotton fabric by about 25–30% (Figure 6a). It is evident that highly efficient cross-linking of the cotton yarn fibers by TPMTI leads to the formation of somewhat brittle short-chain polyurethane layers linking the fibers. Mechanical properties of the Mil-Spec NYCO fabric, which was approximately 2.9-fold stronger than the C2 cotton fabric, were not affected by the treatment via either TPMTI or GPTMS process (Figure 6b). Notably, coating of the cotton C2 or NYCO fabrics via the sol–gel pad–dry process with GPTMS did not change the breaking force or apparent elongation of the coated and halogenated fabrics appreciably. GPTMS and its analogue 3-glycidoxypropyltriethoxysilane are among the most frequently applied silica precursors for hybrid silica-based textile finishing,^54−57^ which form extended cross-links between the silanol groups of the alkoxysilane network and promote adhesion through the epoxy-ring opening with the reactive polymers. The length of the polymer subchain between covalent cross-links defines the mechanical properties of the polymer networks incorporated into the textile finish and hence affects the fabric properties. We can conclude that *process A* involving GPTMS was advantageous over *process B* involving TPMTI from the standpoint of mechanical properties of the resulting coated fabrics. Moreover, from the viewpoint of the potential textile finishing scaleup and commercialization, silica-based organic–inorganic finishes, such as in *process A* (Figure 3), can be considered a promising alternative eco-friendly candidate to isocyanates producing functional textiles. On the other hand, a broad range of multifunctional isocyanates, including eco-friendly ones, are available, which could be applied to optimize the textile finishing process and mechanical properties of the finished textile.^58^

#### 3.3.3 Contact Angle and Wetting by Water

Surface hydrophilicity and wetting characteristics of the expected biocidal surfaces are important in applications ranging from cleaning cloths to air filters and were thus characterized using the sessile drop method. The glass fiber (G) as well as C1 and C2 cotton fabrics were completely wetted by water, which is common and well-known for cotton textiles.^59^ Contact angle on glass fabric was 0°, enabling rapid wicking and water absorption irrespective of the modification by the polymers of the present study. Contact angle of water on the lint-free C1 fabric typically used in cleaning applications was studied in more detail. Treatment by rigid aromatic TPMTI made the surface of the C1 coupons more hydrophobic, with advancing CA in the 120–130° area (Table S2). It was apparent that the polymer solution droplets deposited by spraying from the aqueous solutions onto the TPMTI-treated fabric wicked slowly into the fabric surface. However, after the deposition of the water-soluble polymers and drying/curing, the contact surface angles below 45° showed hydrophilicity of the coupon surfaces, indicating the successful deposition of the hydrophilic polymer chains. The droplets were unstable and wicked into the surface after a few minutes, indicating even higher levels of hydrophilicity than those measured. Bromination or chlorination of the polymer-coated fabric increased the CA by 20–25° (Table S2). When halogenated coupons were rinsed with deionized water and dried under vacuum, the CA on the washed coupons was lowered below the 45° range. The >N–X dissociation and its conversion to the >N–H group of the polymer results in higher hydrophilicity. In fact, a typical halogen bond donor site is significantly less hydrophilic than a hydrogen bond donor site (e.g., an >N–H group).^60^ These experiments show that deposition of hydrophilic polymers followed by halogenation should not interfere with the fabric’s ability to absorb water.

#### 3.3.4 Virucidal Properties

Our previous study demonstrated that when polycationic polymers with guanidine, biguanide, or 4-aminopyridine groups are halogenated and thus converted to N-halamines, they strongly inhibit human respiratory coronavirus.^18^ These findings provided the rationale for the antiviral activity tests of the fabrics coated with such polymers shown in Table 1. Fabrics modified with PHMB had an excellent effect level (AAV > 3) against HCoV-229E, whereas antiviral activity of PAH and PVG was modest (AAV < 3); PVAm coating was not virucidal. The antiviral activity of all polymer-coated fabrics increased dramatically with their halogenation, with all halogenated fabric species exhibiting complete virus inactivation (Inactivation rate, >99%) except for C2-GPTMS-PVAm-Cl (IR = 70%). The time of inactivation of 0.5 h corresponds to the initial period of oxidizing halogen release (compare with Figure 5a), and these results correlate with the hypothesis that PVAm (devoid of biocidal properties prior to halogenation) possesses a lower N–X group dissociation constant than other polymers. It is also possible that the transfer rate of X^+^ from PVAM-X onto microorganisms, such as HCoV-229E, is the lowest among other polymers studied. The complete inactivation of the coronavirus by the halogenated polymer-coated fabrics at 0.5 h where the released halogen concentration is very low (compare with Figure 5a) indicates that the inactivation was realized through the on-contact killing mechanism, wherein the halogen transfer occurred directly from the fabric to the microorganism surface. Such nonleaching on-contact action is desirable for microbicidal textiles.^61,62^

**Table 1:** Activation Activity Value and Inactivation Rate of Human Coronavirus 229E by Cotton (C1 and C2), Nylon–Cotton (NYCO) and Glass Fiber (G) Fabrics Modified by PHMB, PVG, PVAm, and PAH Polymers (5 mg/g Fabric)^a^

material	activation activity value (AAV)	inactivation rate (IR,%)
C1 untreated	0.2	<4
C1-TPMTI-PHMB	5.1	90
C1-GPTMS-PHMB	5.0	88
C1-TPMTI-PHMB-Br	5.6	>99
C1-GPTMS-PVG	2.3	40
C1-GPTMS-PVG-Br	5.6	>99
C1-GPTMS-PAH	2.8	49
C1-GPTMS-PAH-Br	5.6	>99
C1-GPTMS-PVAm	0.2	<4
C1-GPTMS-PVAm-Br	5.6	>99
C2 untreated	0.2	<4
C2-GPTMS-PVAm-Br	4.2	75
C2-GPTMS-PHMB-Br	5.6	>99
C2-GPTMS-PVAm-Cl	4.0	70
C2-GPTMS-PHMB-Cl	5.6	>99
C2-GPTMS-PVG-Cl	5.6	>99
NYCO untreated	0.2	<4
NYCO-GPTMS-PHMB-Br	5.6	>99
NYCO-GPTMS-PHMB-Cl	5.6	>99

a Two-step modification procedures with isocyanate (TPMTI) or bifunctional organosilane (GPTMS) were applied. Time of inactivation: 0.5 h.

Importantly, the coated fabrics can be reused. Fabric coupons modified with PHMB-Br, PVG-Br, and PAH-Br were recovered in triplicate from the virus inhibition studies (above), washed, and steam-sterilized at 121 °C for 30 min. Following sterilization, the fabric coupons were subjected to the halogenation procedure (see Experimental Section). The resulting recharged fabric coupons were subjected to the coronavirus 229E inactivation tests as described above, and all recharged coupons exhibited a >99% coronavirus inactivation rate. To summarize, our polymer-modified and halogenated textile materials exhibit a rapid, durable, and potentially renewable (rechargeable) antiviral activity.

## 4 Concluding Remarks

In this work, we demonstrated that polycationic, biocidal polymers rich in amine, biguanide, guanidine, and hydantoin groups can be covalently attached to a variety of hydrophilic fibrous materials with the surfaces rich in hydroxyl groups by the functional pad–dry–cure finishing using epoxy-functional silane (3-glycidoxypropyltrimethoxysilane) (GPTMS) or by the surface activation using multifunctional isocyanate, followed by covalent grafting of the polymer forming poly(urethane-urea) links with the fabric surface. The pad–dry–cure technique can be scaled using industry-accepted stenter machines, whereas conventional rollcoaters can be used to apply liquid isocyanate adhesive as well as aqueous polymer solutions described in this study. The resulting coatings were nonleaching and maintained their biocidal properties against *S. aureus* and *E. coli* in multiple laundering cycles. The breaking force or apparent elongation of the halogenated woven fabric polymer-coated via the sol–gel pad–dry process with GPTMS did not change. The fabric surfaces coated with water-soluble polymers followed by halogenation remained hydrophilic. Halogenation of the polymer-coated fabrics by aqueous solutions of sodium hypochlorite or by in-situ-generated hypobromous acid resulted in polymer-coated fabrics that contained large loads of halogen. Total chlorine and bromine contents in the fabrics varied in the 0.15–4 and 2–20 mg/g ranges, respectively. The halogen was released slowly when the fabrics were immersed in aqueous phosphate buffer due to the hydrolysis of the N-halamine bonds, but the fabrics were proven to be completely rechargeable with the halogen contents restored. The fabrics coated with halogenated PHMB, PVG, and PAH inactivated respiratory coronavirus 229E completely in 0.5 h tests, demonstrating a rapid, durable, and rechargeable antiviral activity.

## Supporting Information Available

Polymer deposition stability after standard laundering tests; FTIR spectra of the unmodified PHMB and cotton fabric modified by TPMTI; effect of laundering on bactericidal properties of NYCO fabrics; and water contact angles of C1 cotton fabric (PDF)
