# Supplementary material for: Nonleaching Biocidal N-Halamine-Functionalized Polyamine-, Guanidine-, and Hydantoin-Based Coatings
Source: Ind Eng Chem Res. 2024 Mar 27;63(14):6268–78. doi: 10.1021/acs.iecr.4c00320 (PMC11010268; doi:10.1021/acs.iecr.4c00320)
Supplement: Supplementary file 1 — ie4c00320_si_001.pdf [file ie4c00320_si_001.pdf]

SUPPORTING INFORMATION

Non-Leaching Biocidal N-Halamine-Functionalized Polyamine, Guanidine, and Hydantoin-Based Coatings

Lev Bromberg[a], Beatriz Magariños[b], Angel Concheiro[c], T. Alan Hatton[a], Carmen Alvarez-Lorenzo[c,]*

[a]Department of Chemical Engineering, Massachusetts Institute of Technology, Cambridge, MA 02139, USA

[b]Department of Microbiology and Parasitology, Facultad de Biología, CIBUS, Universidade de Santiago de Compostela, 15782 Santiago de Compostela, Spain.

[c]Department of Pharmacology, Pharmacy and Pharmaceutical Technology, I+D Farma Group (GI-1645), Facultad de Farmacia, Instituto de Materiales (iMATUS), and Health Research Institute of Santiago de Compostela (IDIS), Universidade de Santiago de Compostela, 15782 Santiago de Compostela, Spain.

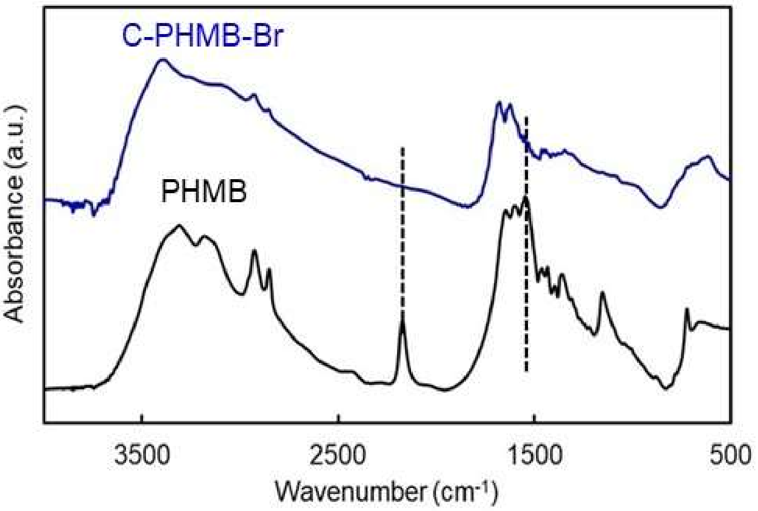

Figure S1. FTIR spectra of the unmodified PHMB and cotton fabric modified by TPMTI and by PHMB, followed by subsequent bromination (C-PHMB-Br). Vertical lines at 1536 and 2170 cm^{-1} indicate scissoring vibration of the N–H and stretching vibration of the terminal -C≡N bond, respectively, present in the unmodified PHMB.

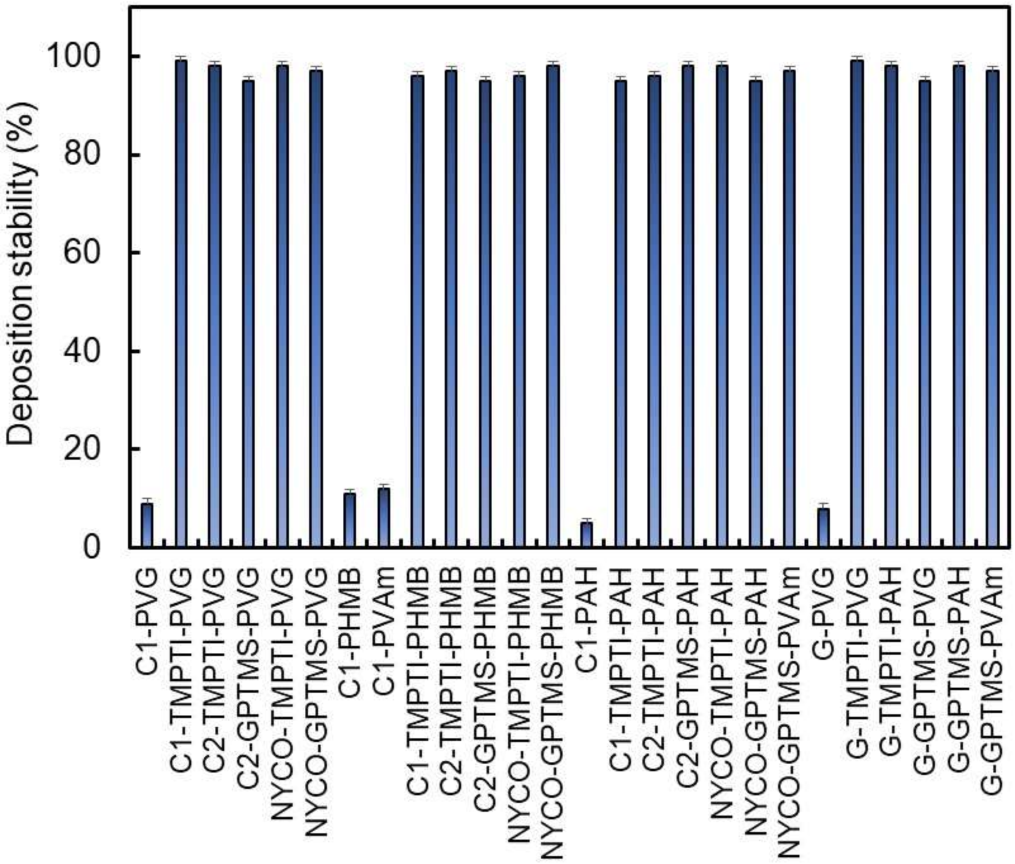

Figure S2. Deposition stability (DS,%) of the polymers under study on glass fiber (G), cotton (C1 and C2) and nylon-cotton (NYCO) fabrics after 15 standard laundering cycles. Designations that include GPTMS indicate that the corresponding fabric was coated via *Process A* using (3-glycidoxypropyl)trimethoxysilane (GPTMS), whereas designations that include TPMTI indicate that the corresponding fabric was coated via *Process B* using (3- triphenylmethane-4,4',4''-triisocyanate (TPMTI). Samples designated as C1-PVG, C1-PHMB, C1-PAH, G-PVG were prepared by a simple adsorption of the corresponding polymers via spraying polymer solutions following drying at 45ºC on air until constant weight.

Table S1. Effect of laundering on bactericidal properties of PHMB-modified C2 and NYCO fabrics. Bactericidal activity of cotton C2 and NYCO fabrics coated by PHMB against *S. aureus* and *E.coli* after 15-cycle laundering. Designations C2-GPTMS-PHMB and NYCO-GPTMS-PHMB indicate that the corresponding fabric was coated via *Process A* using (3-glycidoxypropyl)trimethoxysilane (GPTMS), whereas designations C2-TPMTI -PHMB and NYCO-TPMTI -PHMB indicate that the corresponding fabric was coated via *Process B* using (3-triphenylmethane-4,4',4''-triisocyanate (TPMTI). The antimicrobial activity was expressed as *Reduction (%) =100 x P/Q*, where *P* and *Q* are the numbers of surviving colonies in the flasks containing the polymer-coated and control samples, respectively.

**Fabric**	***S. aureus***		***E. coli***	
	Surviving cells, 10^{-5} x cfu/mL [a]	Reduction, %	Surviving cells, 10^{-5} x cfu/mL [b]	Reduction, %
C2-GPTMS-PHMB	4.95	96.7	5.76	95.8
C2-TPMTI-PHMB	5.1	99.6	5.91	98.3
NYCO-GPTMS-PHMB	20.2	95.0	30.5	96.9
NYCO-TPMTI-PHMB	20.3	95.1	30.8	97.8

[a] Surviving cells reading for C2 untreated and NYCO fabric untreated (control) were 5.12 x10^5 and 21.3x10^5 cfu/mL, respectively, for *S. aureus*.

[b] Surviving cells readings for C2 untreated and NYCO fabric untreated (control) were 6.01 x10^5 and 31.5x10^5 cfu/mL, respectively, for *E. coli.*

Table S2. Water contact angles of C1 cotton fabric coated by polymers of the current study, before and after halogenation.

**Cotton fabric treatment**	**Advancing contact angle (°)**
Untreated	16-19
TPMTI	120-128
TPMTI-PHMB	37-43
TPMTI-PHMB-Br	57-60
TPMTI-PVG	40-43
TPMTI-PVG-Br	69-74
TPMTI-PVG-Cl	64-72
TPMTI-PAH	35-39
TPMTI-PAH-Br	58-65
TPMTI-PAH-Cl	50-61
GPTMS	24-30
GPTMS-PAH-Br	45-51
